# Supplementary material for: Asynchronous magnetic resonance elastography: Shear wave speed reconstruction using noise correlation of incoherent waves
Source: Magn Reson Med. 2022 Oct 27;89(3):990–1001. doi: 10.1002/mrm.29502 (PMC9792433; doi:10.1002/mrm.29502)
Supplement: Supplementary file 1 — DATA S1 MATLAB code used to generate the numerical simulations of Figure 1 [file MRM-89-990-s002.zip › k-Wave/helpfiles/getDateString.html]

getDateString :: Functions (k-Wave)


# getDateString

Create a string of the current date and time.

## Syntax

```
date_string = getDateString()
```

## Description

`getDateString` returns a string of the current date and time using `datestr` but replacing the white space and : character with an en dash.

## Outputs

|  |  |
| --- | --- |
| `date_string` | formatted string of the current date and time |

## See Also

`clock`, `date`, `datestr`
